# 本科生综合实验:基于共价有机框架的核壳型固定相的制备及色谱性能评价

**DOI:** 10.3724/SP.J.1123.2024.03003

**Published:** 2024-10-08

**Authors:** Jin LIU, Wenchang TIAN, Fan WU, Qiuting ZHANG, Yanhui ZHONG, Zian LIN

**Affiliations:** 福州大学化学学院, 食品安全与生物分析教育部重点实验室, 福建省食品安全分析与检测重点实验室, 福建 福州 350108; Ministry of Education Key Laboratory of Analytical Science for Food Safety and Biology, Fujian Provincial Key Laboratory of Analysis and Detection Technology for Food Safety, College of Chemistry, Fuzhou University, Fuzhou 350108, China

**Keywords:** 综合性实验, 共价有机框架, 色谱固定相, 核壳结构, comprehensive experiment, covalent organic framework, chromatographic stationary phase, core-shell structure

## Abstract

在教育部不断推进新课程标准的背景下,本文介绍了一项面向本科生仪器分析课程的综合性教学实验,旨在通过实践提高学生在材料制备和仪器使用方面的技能。该实验通过“一锅法”制备了一种基于共价有机框架的核壳结构固定相材料(SiO_2_@COF_TTA-DHTA_),并对其进行了详细的理化表征。随后,采用高效液相色谱技术对该材料的色谱性能进行了评估。整个实验流程包括了材料合成、表征、色谱保留行为研究和色谱分离性能测试。该实验不仅加深了学生对于功能材料性质及其应用的理解,而且提高了他们的实验设计和批判性思维能力。通过理论科学与实验教学的结合,该实验不仅能够培养学生的科研兴趣,还可以锻炼实验操作能力、创新思维和实践能力,同时增强学生的社会责任感和历史使命感,实现实验教学的全方位育人目标。

## Abstract (en)

Continuously promoting new curriculum standards is a key aim of the Ministry of Education of China. With this in mind, this paper introduces a comprehensive teaching experiment for undergraduate instrumental analysis courses that aims to improve students’ material-preparation and instrumental-analysis skills through practice. Herein, a covalent organic framework-based core-shell stationary phase material (SiO_2_@COF_TTA-DHTA_) is prepared via a one-pot method and characterized in detail, after which its chromatographic properties are evaluated by high performance liquid chromatography (HPLC). The experimental process includes material synthesis and characterization, as well as studying the chromatographic-retention behavior and chromatographic-separation performance of the material. By the combining theoretical science and experimental teaching, this experiment not only deepens students’ understanding of the properties of functional materials and their applications, but also improves their experimental-design and critical-thinking skills. This experiment not only cultivates students’ interests in scientific research, but also exercises their experimental, operational, innovative-thinking, and practical abilities, while concurrently enhancing their sense of social responsibility and historical mission, thereby delivering the all-round educational goals of experimental teaching.

仪器分析是一门实践性与应用性极强的学科,在化学、药学、生物和食品等领域发挥着重要的作用。仪器分析教学以教材理论为核心,以实践应用为目标,旨在培养学生习得扎实的仪器理论知识并能将其灵活应用到实践中,从而加强学生利用各类大型仪器解决问题的能力,实现实验教学培育人才的课程目标。本教学团队基于共价有机框架(COFs)材料和色谱分离技术的研究,将科研成果转化为教学资源,设计了本科生综合性实验,从而激发学生的科研兴趣,锻炼实验操作能力,并培养创新和实践能力。

色谱法是一种物理化学分析方法,已被广泛应用于分离领域。高效液相色谱(HPLC)作为最常用的色谱分离方法,是仪器分析学科中的重点学习内容^[1]^。HPLC具有效率高、灵敏度好、操作方便等优点,被广泛应用于食品分析^[2]^、药物分析^[3]^、环境分析^[4]^等领域。HPLC仪由5个部分组成,分别为高压输液系统、进样系统、分离系统、检测系统和记录系统。其中色谱柱是HPLC的关键部件。而色谱柱的填料,即色谱固定相,作为色谱分离系统的“核心”,直接决定着色谱分离的性能。传统硅胶固定相制备工艺复杂、不耐受酸碱;聚合物固定相虽具有较宽的pH适用范围,但其机械稳定性较差且易于溶胀。因此,新型色谱固定相的开发一直是色谱工作者研究的前沿和热点之一^[5]^。COFs因具有独特的结构、高比表面积、高孔隙度及优异的热稳定性和化学稳定性,在色谱分离中显示出巨大的应用潜力^[6]^。因此,本实验将COFs与传统固定相材料二氧化硅(SiO_2_)微球相结合,制备出一种新型色谱固定相(SiO_2_@COF),并对其进行物理化学表征,最后将其应用于色谱分离中,考察其色谱保留机理和色谱分离性能。

本科阶段的仪器分析实验主要引导学生理论联系实际,启发学生思维,调动学生的学习积极性和主动性,增强学生独立分析问题和解决问题的能力^[7]^。体现在化学学科上,即要充分发挥化学是一门实验性学科的特点。在HPLC技术方面,本校已开设一些基础类教学实验,基本循序了经典的实验内容,但前沿性与创新性相对欠缺。因此,我们选择将新型色谱固定相的制备及色谱性能评价作为本科实验教学项目,引入液相色谱固定相制备、表征、装填及分离性能评价等内容,涵盖了有机化学、物理化学和仪器分析等多学科知识。学生通过文献调研、分组讨论和实验操作,不仅可以拓宽知识面,还能够充分提升对于仪器分析实验的学习兴趣,有利于培养学生对知识的综合运用能力和对科学研究的钻研探索能力^[8]^。

## 1 实验部分

### 1.1 实验目的

(1)掌握液相色谱仪的基本结构、工作原理和基础操作。(2)掌握色谱动力学基础理论(包括塔板理论及速率理论)。(3)了解液相色谱固定相的制备方法,色谱柱填充的基本流程和性能评价方法。

### 1.2 实验原理

HPLC根据混合物中各组分在固定相和流动相之间分配比例的不同而实现各组分的分离^[9]^。我们选用了氨基修饰的二氧化硅微球(SiO_2_-NH_2_),这些氨基是二氧化硅微球能够进行表面化学修饰和改性的基础。通常二氧化硅微球上的氨基可以与醛氨缩合形成的COFs上未反应的醛基发生席夫碱反应,从而使得COFs能够修饰到二氧化硅微球上形成SiO_2_@COF复合材料。

色谱动力学理论是根据流体分子运动规律研究色谱过程中分子迁移与分离的机制,其主要包括两个重要的理论,分别是塔板理论和速率理论^[9]^。

塔板理论是将色谱柱看作一个分馏塔,设想其中均匀分布许多塔板,当待分离组分从分馏塔的每个塔板流下并达到平衡,最终流出液峰形对称且呈正态分布时,通过公式(1)来计算理论塔板数(*N*)。


(1) $$N=5.54\times {\left(\frac{t_{R}}{W_{1/2}}\right)}^{2}$$

式中,*t*_R_表示分析物的保留时间,*W*_1/2_表示流出物质的半峰宽。通过比较不同组分的理论塔板数就可以得出色谱柱的柱效。

速率理论概括了色谱峰扩展的各种基本因素。速率理论方程又名van Deemter方程,是对塔板理论的修正,用于解释色谱峰扩张和柱效降低的原因。其表达式如下:


(2) $$H=A+\frac{B}{u}+Cu$$

式中,*H*为塔板高,*u*为流动相平均线速度,*A*为涡流扩散因素,*B/u*为分子扩散因素,*Cu*为传质因素。*A*项描述了色谱柱中固定相颗粒引起的峰展宽。该效应与填料的粒径、形貌、孔径的形状及结构有关。填料粒径越大、形状越不规则、孔径的形状与结构越不规整,溶质分子的多路径效应越为明显,柱效越低,峰展宽越严重。*B*项代表溶质在色谱柱流动中的“纵向或被动扩散”,其大小取决于绝对温度、分子的大小、流体的黏度以及分子的流速。流速减小或温度升高时,扩散系数变大,扩散通量也变大,纵向扩散效应更为明显,相反,流速增大或温度降低时,扩散系数变小,扩散通量也变小,纵向扩散效应则不明显。*C*项与分离过程中样品组分在固定相和流动相之间的传质有关。流动相中的溶质分子存在与流动相相同的速度场分布,而不同的流速分布就造成了溶质分子传质过程的差异——色谱柱中心的溶质分子传质速率越快,距离色谱柱中心越远则传质速率越慢,从而导致流动相中溶质分子峰展宽。

此外,采用保留因子(*k*)、分离度(*R*_s_)作为色谱性能评价参数,计算公式如下:


(3) $$k=\frac{t_{R}-t_{0}}{t_{0}}$$

(4) $$R_{s}=\frac{t_{R2}-t_{R1}}{W_{V/2(1)}-W_{1/2(2)}}$$

式中,*t*_0_为色谱柱的死时间,*t*_R1_和*t*_R2_分别为第一个和第二个流出物质的保留时间, ${W_{l/2}}_{\left(1\right)}$ 和 ${W_{1/2}}_{\left(2\right)}$ 分别为第一个和第二个流出物质的半峰宽。

### 1.3 仪器与试剂

HY-HPLC-M色谱装柱机(北京海德利森科技公司), LC 20AT MS高效液相色谱仪(Shimadzu,日本), ASAP 2020物理吸脱附仪(BET, Micromeritics,美国), X’Pert Pro MPD多功能X射线衍射仪(XRD, Philips,荷兰), Nicolet iS50傅里叶变换红外光谱仪(FTIR, Thermo,美国), SU8020扫描电子显微镜(SEM)和HT7700透射电子显微镜(TEM)(Hitachi,日本)。

所有化学试剂均为分析纯及以上纯度,无需进一步纯化处理。SiO_2_-NH_2_购自天津市倍思乐色谱技术开发中心。 2,4,6-三(4-氨基苯基)-1,3,5-三嗪(TTA, 98%)和2,5-二羟基对苯二甲醛(DHTA, 99%)购自吉林中科研伸科技有限公司。醋酸、甲醇、乙腈(ACN)、四氢呋喃(THF)、乙醇、异丙醇、正己烷、丙酮、二氧六环和均三甲苯购自国药化学试剂有限公司。苯胺类(苯胺(96%)、四乙烯基苯胺(95%)、萘胺(97%)、二苯胺(95%))、烷基苯类(甲苯(96%)、乙苯(95%)、丙苯(95%)、丁苯(97%))、多环芳烃(PAHs)类(苯(98%)、萘(97%)、芴(95%)、菲(98%)、联苯(96%)、苊(98%)、对三联苯(96%)、三亚苯(95%))购自上海阿拉丁试剂有限公司。醌类(1,4-萘醌(95%)、苊醌(97%)、菲醌(95%)、蒽醌(96%)、2-甲基蒽醌(95%))购自上海麦克林生化科技有限公司。

### 1.4 实验步骤

#### 1.4.1 SiO_2_@COF_TTA-DHTA_的制备

实验流程图如图1所示。首先将TTA(21 mg, 0.02 μmol)、DHTA(21 mg, 0.03 μmol)和SiO_2_-NH_2_(50 mg)超声溶解于均三甲苯/二氧六环(2 mL, 1.7∶0.3, v/v)中。随后将上述溶液加入到10 mL耐热管中,并加入150 μL醋酸(17.5 mol/L)。然后通过液氮快速冷冻耐热管并用真空泵抽取空气,连续操作3次。最后等待耐热管恢复至室温,放入120 ℃烘箱中反应3天。将得到的产物离心,并用THF和乙醇洗涤数次。将洗涤后的产物置于60 ℃真空下干燥过夜备用。

**图1:**
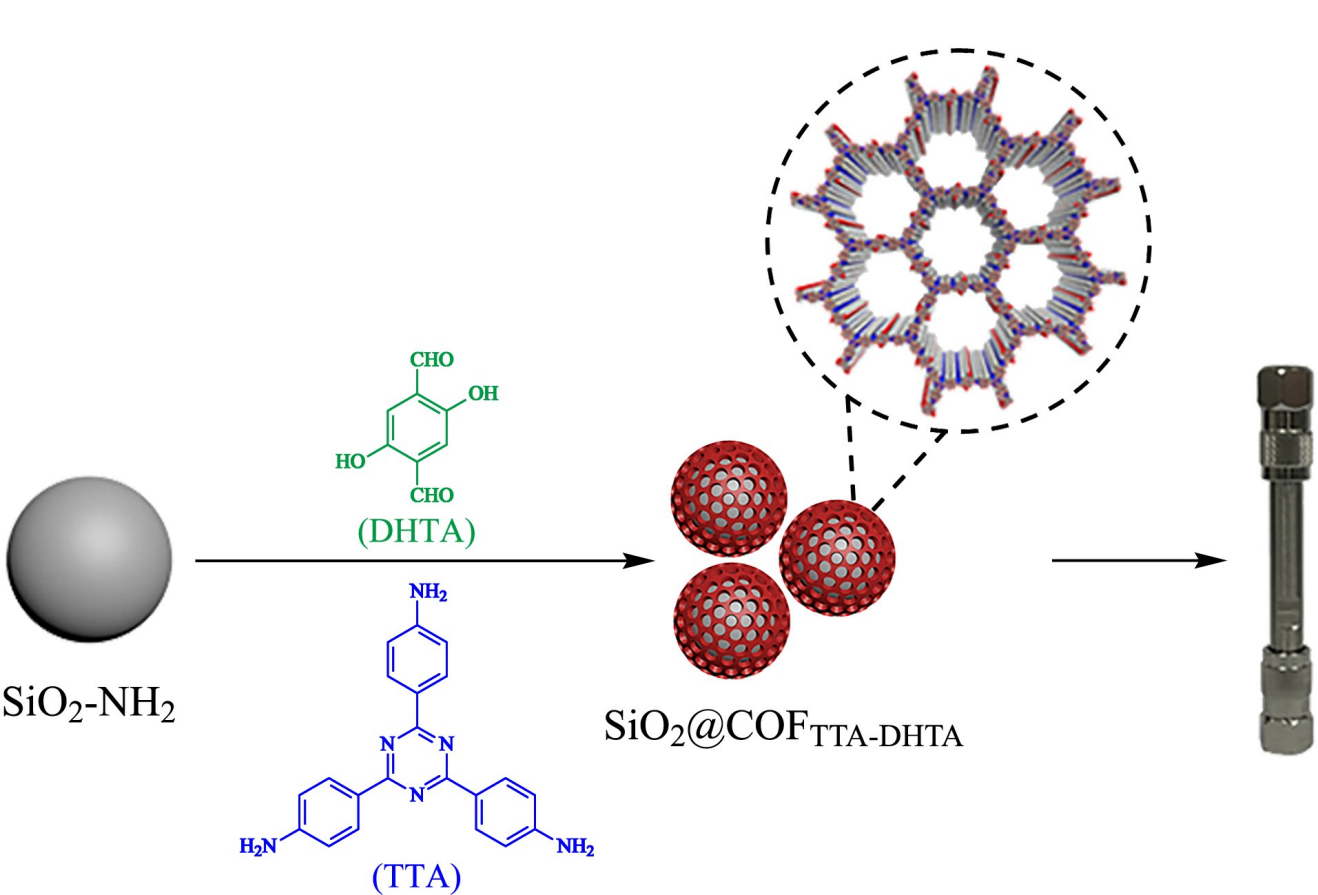
SiO_2_@COF_TTA-DHTA_填充柱的制备流程

#### 1.4.2 色谱柱的装填

所制备的核壳型色谱固定相采用高压匀浆法填充到不锈钢色谱柱管(50 mm×2.1 mm i. d.)中。匀浆液为体积比1∶1的异丙醇和乙腈混合液,顶替液为甲醇,装柱机装填压力为34.5 MPa (5000 psi)。SiO_2_-NH_2_填充柱的装填方法同上。

#### 1.4.3 实验条件

分别称取烷基苯类、PAHs类、苯胺类和醌类标准品各10 mg溶解于10 mL乙腈中,得到1 g/L的母液。量取苯母液200 μL、萘母液50 μL和1,4-萘醌母液100 μL,用乙腈稀释至1 mL得到烷基苯类物质进样液。其他各类物质分别量取母液各100 μL,用乙腈稀释至1 mL,得到相应的进样液。

色谱条件:流动相为乙腈-水(ACN/H_2_O),烷基苯类、PAHs类流动相为ACN/H_2_O(70∶30, v/v),苯胺类流动相为ACN/H_2_O(50∶50, v/v),醌类流动相为ACN/H_2_O(60∶40, v/v);流速为0.3 mL/min;进样量为5 μL;柱温为35 ℃;烷基苯类、苯胺类和醌类物质的检测波长为214 nm,多环芳烃类物质的检测波长为210 nm。

#### 1.4.4 实际样品分析

醌类是可吸入颗粒物(PM_2.5_)中的一种重要的致病因子,因此对PM_2.5_中的醌类物质进行分离分析是评价其健康风险的基础。本实验中的PM_2.5_样品由山西省环保部门提供(2019年12月)。样品制备方法如下:首先将60 cm^2^的PM_2.5_样品滤膜分割成条状,再放置于50 mL烧杯中,加入20 mL正己烷/丙酮(1∶1, v/v),超声1 h。将超声后的溶液转移至烧瓶,用旋转蒸发器蒸干,加入2 mL乙腈润洗烧瓶并过滤溶液至离心管中,即为PM_2.5_样品溶液。以PM_2.5_样品溶液稀释醌类标准溶液,即得PM_2.5_加标样品溶液。

## 2 结果与讨论

### 2.1 材料的表征

在FTIR谱图中,465 cm^-1^和1088 cm^-1^处的特征拉伸振动归因于SiO_2_-NH_2_的Si-O和Si-O-Si的振动,1626 cm^-1^处的特征拉伸振动归因于COFs的C=N振动。可以清楚地看到,核壳结构的SiO_2_@COF_TTA-DHTA_光谱中出现了SiO_2_-NH_2_和COFs的特征吸收峰,证明COFs成功修饰到了SiO_2_-NH_2_表面(图2a)。如图2b所示,SiO_2_@COF_TTA-DHTA_微球的结晶度与COFs一致,表明COFs修饰到SiO_2_-NH_2_表面后其晶体结构并未遭到破坏。SEM和TEM分析了SiO_2_-NH_2_和核壳结构SiO_2_@COF_TTA-DHTA_微球的形貌。如图2c和2d所示,与SiO_2_-NH_2_相比,SiO_2_@COF_TTA-DHTA_微球的表面更为粗糙,表明COFs成功固载在SiO_2_-NH_2_表面。TEM图像(图2e和2f)中,SiO_2_@COF_TTA-DHTA_微球呈现出独特的核壳形态,壳层COF_TTA-DHTA_的厚度约为200 nm。

**图2:**
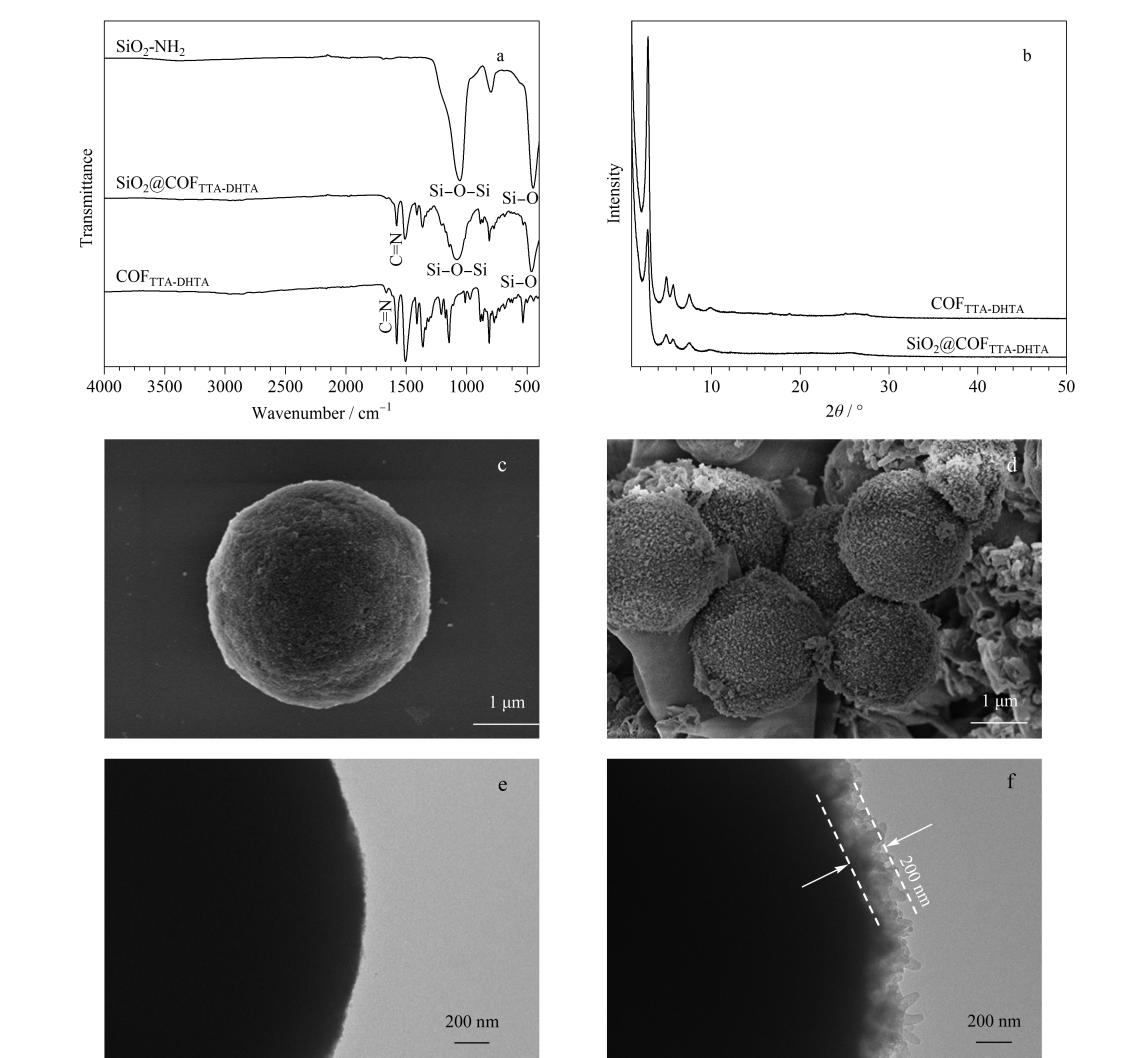
(a)SiO_2_-NH_2_、SiO_2_@COF_TTA-DHTA_和COF_TTA-DHTA_的红外光谱图;(b)COF_TTA-DHTA_和SiO_2_@COF_TTA-DHTA_的粉末X射线衍射 (PXRD)图;(c)SiO_2_-NH_2_和(d)SiO_2_@COF_TTA-DHTA_的SEM图;(e)SiO_2_-NH_2_和(f)SiO_2_@COF_TTA-DHTA_的TEM图

### 2.2 色谱保留机理探究

由于SiO_2_@COF_TTA-DHTA_核壳型固定相含有多个苯环,可以推测SiO_2_@COF_TTA-DHTA_填充柱在分离过程中提供疏水相互作用。所以选择非极性烷基苯类物质作为研究对象,来探究SiO_2_@COF_TTA-DHTA_填充柱的保留机理。结果显示,随着ACN的含量从40%增加至60%, 4种烷基苯类物质的保留时间逐渐减少,呈现出典型的反相液相色谱(RPLC)保留模式(图3)。

**图3:**
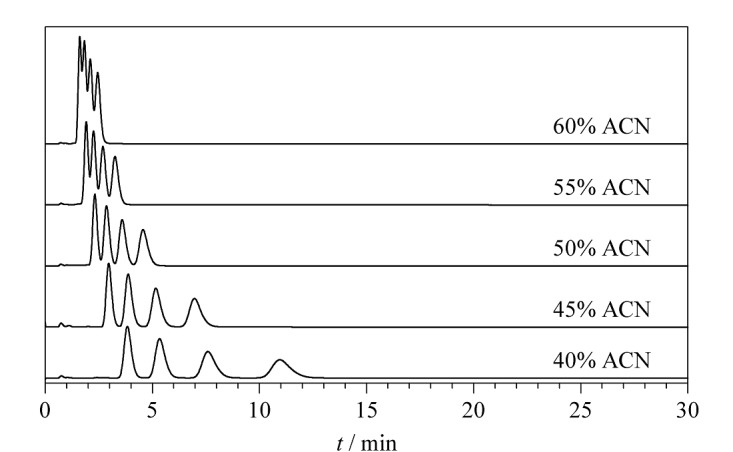
流动相中ACN含量对烷基苯类物质分离的影响

### 2.3 色谱性能考察

#### 2.3.1 多环芳烃类物质的分离

首先,选取一组PAHs类物质(苯、萘、芴和菲)来考察SiO_2_@COF_TTA-DHTA_填充柱的分离性能。由图4a可以看出,在ACN/H_2_O(70∶30, v/v)的流动相条件下,4种PAHs分析物在SiO_2_@COF_TTA-DHTA_填充柱上均实现了基线分离,且4种物质的洗脱顺序(苯、萘、芴、菲)遵循物质的疏水性强弱顺序。在相同的分离条件下,SiO_2_-NH_2_填充柱无法分离上述4种物质,进一步证明COF_TTA-DHTA_在PAHs类物质的分离过程中起主导作用。由结构可知,SiO_2_@COF_TTA-DHTA_核壳型固定相含有多个苯环,可与PAHs类物质形成*π-π*相互作用,改善固定相的分离性能。因此,选取另外一组共轭结构不同的PAHs类物质(联苯、苊、对三联苯、三亚苯)继续考察其分离性能。所获得的结果如图4b所示,4种PAHs的洗脱顺序(联苯、苊、对三联苯、三亚苯)与其共轭强弱顺序一致,证明PAHs与SiO_2_@COF_TTA-DHTA_固定相之间还存在*π-π*相互作用。以上结果表明,SiO_2_@COF_TTA-DHTA_填充柱对多环芳烃具有良好的分离效果。根据公式(1)计算出联苯、苊、对三联苯和三亚苯在SiO_2_@COF_TTA-DHTA_填充柱上的柱效为10580~13420 plate/m。根据公式(4)计算出联苯、苊、对三联苯和三亚苯在SiO_2_@COF_TTA-DHTA_填充柱上的分离度分别为3.27、1.57和2.59。综上所述,PAHs类物质在SiO_2_@COF_TTA-DHTA_填充柱上可实现快速分离。

**图4:**
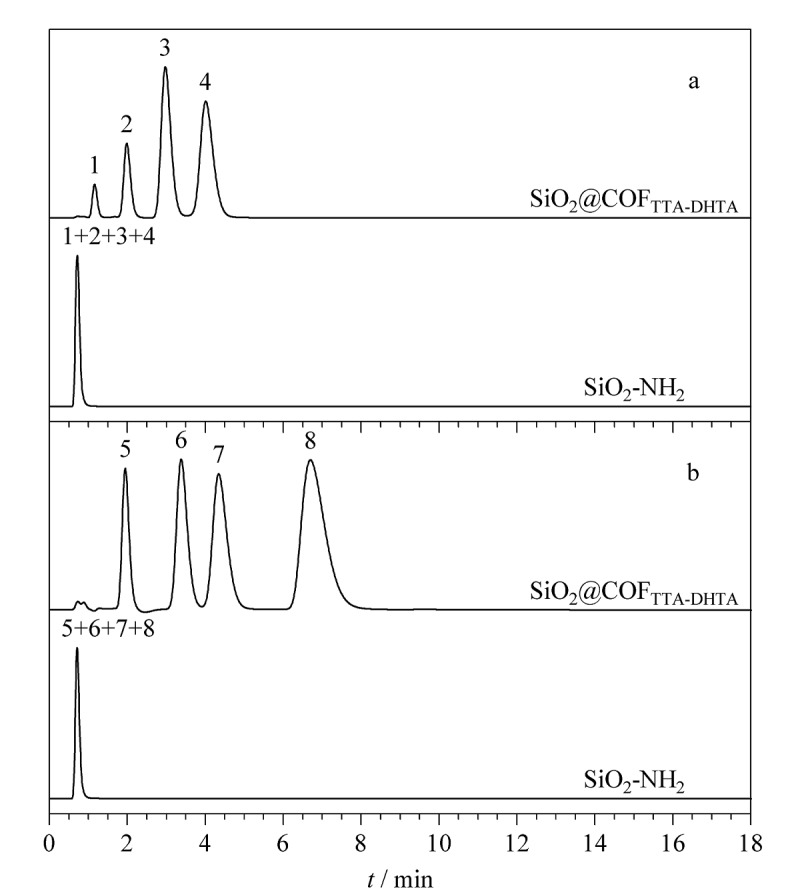
PAHs在SiO_2_@COF_TTA-DHTA_和SiO_2_-NH_2_填充柱上的色谱图

#### 2.3.2 苯胺类物质的分离

鉴于SiO_2_@COF_TTA-DHTA_填充柱对PAHs的优异分离效果,实验选取了一组苯胺类物质(苯胺、四乙烯基苯胺、萘胺和二苯胺)进一步考察SiO_2_@COF_TTA-DHTA_填充柱的分离性能。如图5所示,SiO_2_@COF_TTA-DHTA_填充柱在流动相为ACN/H_2_O(50∶50, v/v)条件下,6 min内即可实现苯胺、四乙烯基苯胺、萘胺和二苯胺的基线分离,且峰形对称,出峰顺序为苯胺、四乙烯基苯胺、萘胺、二苯胺。在同样的分离条件下,SiO_2_-NH_2_填充柱无法分离上述4种物质,进一步证明SiO_2_@COF_TTA-DHTA_在苯胺类物质分离过程中的重要作用。

**图5:**
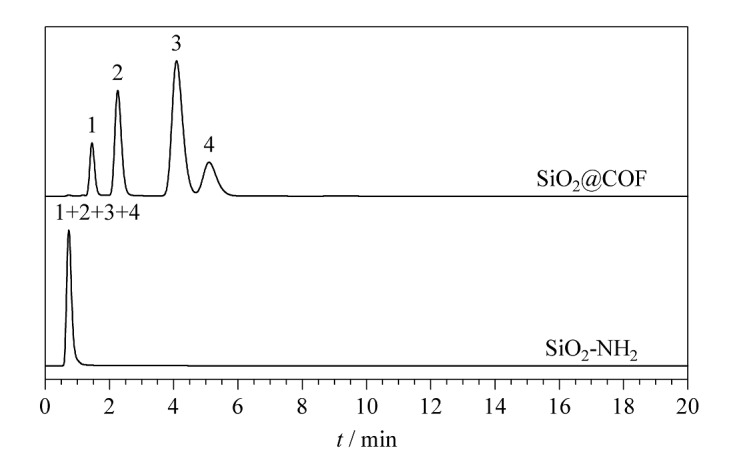
苯类物质在SiO_2_@COF_TTA-DHTA_和SiO_2_-NH_2_填充柱上的色谱图

### 2.4 PM_2.5_中醌类化合物的分离

PM_2.5_是定义为空气动力直径小于或者等于2.5 μm的大气颗粒物。它可以深入肺泡,对人体健康产生威胁^[10]^。现有资料报道,PM_2.5_携带的化学物质(如无机离子、多环芳烃及其衍生物等)具有一定毒性作用^[11]^。PM_2.5_中的醌类物质是一类含氧多环芳烃,主要来源于化学燃料的不完全燃烧^[12]^,因其具有较强的毒性作用而备受关注。实验将SiO_2_@COF_TTA-DHTA_填充柱用于PM_2.5_中醌类物质的分离分析。如图6所示,PM_2.5_中醌类物质在SiO_2_@COF_TTA-DHTA_填充柱上获得了良好的分离效果,且PM_2.5_中的基质对醌类物质的分离未产生干扰。综上所述,SiO_2_@COF_TTA-DHTA_填充柱可用于PM_2.5_中醌类物质的分离。

**图6:**
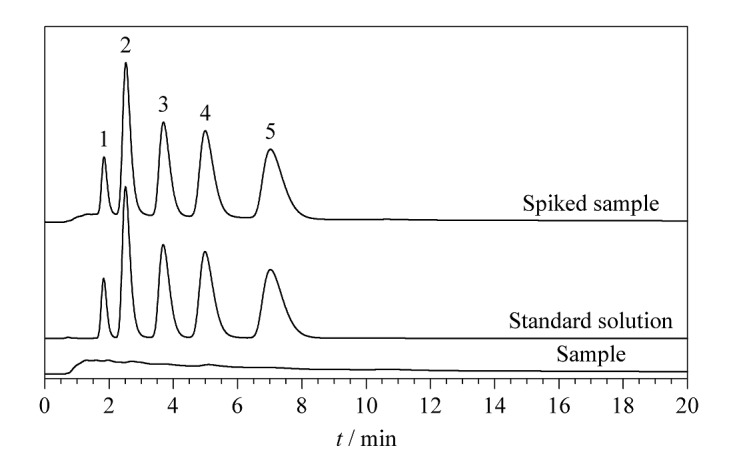
PM_2.5_样品、醌类标准溶液和PM_2.5_加标样品 在SiO_2_@COF_TTA-DHTA_填充柱上的色谱图

## 3 实验安排与教学反思

### 3.1 实验教学安排

本实验采用线下教学方法。实验前,学生需要查阅相关文献,通过线上平台观看演示视频,学习仪器操作,提前了解固定相制备流程和HPLC使用注意事项。学生需要根据预习内容制定实验方案,并与任课老师讨论以优化实验方案,从中锻炼自身独立思考能力与创新思维意识。建议学生2~3人分为一组,明确各自的职责和任务,相互协作,共同完成实验。实验过程共计12学时,包括材料制备3学时、表征3学时、固定相装填3学时、色谱保留机理探究1学时、色谱性能考察与评价1学时、实际样品分析1学时。实验结束后,鼓励每个实验小组对实验结果进行深入讨论,提出改进方案,并协作完成实验报告。教师在教学过程中应秉持“学生为主,教师为辅”的原则,让学生大胆尝试,反复练习,发挥引导和反馈的作用,帮助学生理解不同版块之间的联系。

### 3.2 教学反馈与改进

实验结束后,教师调研了学生对于本次实验的评价。调研结果表明,该实验中色谱分离性能研究部分各类物质分离效果明显,具有一定趣味性和实用性,学生对这一综合性实验给予极高的评价和反响;经历了“文献查阅、材料制备、表征测试到分析应用”整个实验过程后,学生的自主学习能力、解决问题能力以及创新思维意识均有很大提升。此外,这一实验也间接增强了学生对于前沿科学研究的关注。

为进一步提高实验教学质量,计划采取以下改进措施。(1) TEM和SEM操作优化:TEM和SEM制样时,应尽可能保持样品均匀分散于溶剂中,分散后的样品应均匀覆盖在铜网或硅片上,避免过度集中或分散不均。由于TEM和SEM属于大型仪器,需要经专业操作培训人员上机操作,在此学生仅需观看拍摄过程,无需自主操作;(2)本实验需运用HPLC仪等多种可自主操作大型仪器。在仪器使用前,学生应全面预习相关仪器说明,教师授课时应充分讲解仪器原理、操作步骤及注意事项;(3)本实验色谱柱填充过程较为繁琐且较易失败,该部分实验由教师提前录制操作视频,并在课堂进行仔细讲解和演示,同时叮嘱学生填柱过程中的注意事项,以避免不必要的失败;(4)实验开展过程中,可能出现色谱柱柱压不稳定的情况,此时需要使用高有机相对色谱柱进行冲洗,直至柱压稳定后方可继续使用。

## 4 结语

本文介绍了一个仪器分析综合性实验,本实验在本科生《现代化学实验与技术(仪器分析部分)》综合实验课程中具有较高的教育和培养意义。该实验引入固定相的制备、表征手段及色谱分离性能评价等内容,充分体现了化学学科的创造性和实用性。学生不仅可以理解材料特性与色谱应用之间的关系,还可以掌握液相色谱仪的工作原理和使用方法,理解色谱动力学的基础理论。该实验内容系统而丰富,涵盖了多学科知识,综合性与实用性较强。通过系统的文献调研、分组讨论、实验操作、数据处理和实验报告撰写,学生可更加深入和全面地理解色谱学中抽象的理论知识。该实验通过理论科学与实验教学的结合,不断激发学生的科研兴趣,锻炼学生的创新和实践能力,为培养具有国际视野和敏锐洞察力的人才奠定了基础,也响应了教育部所提出的理论与实践相结合、科研与教学相结合的号召。
